# Ranging Behaviour of Verreaux’s Eagles during the Pre-Breeding Period Determined through the Use of High Temporal Resolution Tracking

**DOI:** 10.1371/journal.pone.0163378

**Published:** 2016-10-10

**Authors:** Megan Murgatroyd, Les G. Underhill, Willem Bouten, Arjun Amar

**Affiliations:** 1 Animal Demography Unit, Department of Biological Sciences, University of Cape Town, Rondebosch, 7701, South Africa; 2 Percy FitzPatrick Institute of African Ornithology, Department of Biological Sciences, University of Cape Town, Rondebosch, 7701, South Africa; 3 Institute for Biodiversity and Ecosystem Dynamics, University of Amsterdam, Amsterdam, Netherlands; Universita degli Studi di Milano-Bicocca, ITALY

## Abstract

Information on movement ecology is key in understanding the drivers and limitations of life history traits and has a potential role in indicating environmental change. Currently we have a limited understanding of the parameters of movement of territory-bound raptors, which are sensitive to environmental change. In this study we used GPS tracking technology to obtain spatially (within 3 m) and temporally (*c*. 3 mins) high-resolution movement data on a small sample of Verreaux’s eagle *Aquila verreauxii* during the pre-laying period (*n* = 4) with one additional example during the chick rearing period. We present GPS-derived home range estimates for this species and we examine temporal (timing, duration, frequency and speed) and spatial (total path length and maximum distance from nest) patterns of trips away from the nest. For eagles tagged in the agriculturally developed Sandveld region (*n* = 3), which is made up of a mosaic of land use types, we also undertook a habitat selection analysis. Home ranges were small and largely mutually exclusive. Trip activity was centred around midday, which is likely to be related to lift availability. Our habitat selection analysis found that eagles selected for near-natural and degraded habitat over natural or completely modified areas, suggesting that these eagles may have benefitted from some of the agricultural development in this region. Although our sample sizes are small, the resolution of our tracking data was essential in deriving this data over a relatively short time period and paves the way for future research.

## Introduction

Understanding spatio-temporal patterns of animal activity is the central theme in the growing field of movement ecology. Information on individual animal movement and space use is key in understanding the environmental drivers and the limitations on a species life history traits, as well as forecasting their persistence and implementing conservation strategies in a rapidly changing world [1,2]. The ranging behaviour of a species is often correlated with food availability [3–5]. Land use change often causes the depletion of habitats supporting traditional prey resources [6]. Studies investigating foraging trip parameters in some avian guilds have identified longer duration trips, longer path lengths and greater maximum distances from nest sites as indicators of a greater foraging effort driven by prey declines [7,8]. Likewise, habitat selection is evident in some species, which is usually related to the depletion or abundance of food resources [6,9,10]. As such, changes in movement behaviour might even be an early warning signal of a stressed species. Despite the importance of this field of research, an empirical framework for understanding of daily movement patterns of resident raptors has not yet been reached. The relevance of this for raptors, is particularly important due to their position as an apex predator which i) leaves them susceptible to negative effects of changes in the environment [11] and ii) gives them an important role in structuring the ecosystem [12]. These are both traits which contribute to the role raptors can play as potential indicator species [13] and if the subtleties of their movement ecology, particularly when foraging, can be fully understood this might give a greater insight into the spatiotemporal distribution of prey in their environment and the health of the ecosystem [8,14].

The Verreaux’s eagle *Aquila verreauxii* is a large, territorial raptor which has been well documented as a specialist hunter of hyraxes (*Procavia* spp. and *Heterohyrax* spp.*)* when available [15–17]. It is a year-round resident, and although their nesting sites on steep cliffs are relatively immune to human disturbance, their dependency on a localized prey base and their relatively specialized diet could leave them susceptible to negative impacts of increasing human pressures [15,18,19]. Globally, the Verreaux’s eagle is listed as a species of Least Concern given its extensive range throughout much of eastern and southern Africa [20]. However, within southern Africa the species has recently been classified as “Vulnerable” [21] due to decreases in range and abundance recorded by the Southern African Bird Atlas Projects [22]. For example, decreases in the number of resident pairs on the Cape Peninsula, South Africa, are thought to be related to the loss of prey resources and disturbance caused by urbanization [23]. Reduction of traditional prey resources has also been linked with a diet shift and the need for supplementary feeding of a pair on the outskirts of Johannesburg [19]. On communal lands in Zimbabwe, nest abandonment and reduced breeding productivity have been associated with reduced hyrax numbers due to hunting and increased disturbance [15]. Despite considerable historical research on many aspects of the Verreaux’s eagle ecology [15–17,24–26], there is almost no information available on their movement ecology or behaviour away from the nest site. GPS tracking technologies have enabled data collection on these aspects for several other raptor species [3,4,27]. On-going technological developments in this field are providing progressively higher-resolution data giving unprecedented insights into movement ecology. These advances in the temporal and spatial resolution of data have also been followed by developments of analytical methods [28].

Here, we investigate aspects of Verreaux’s eagle movement ecology using GPS tracking technology. Firstly, we describe the home ranges and their core use by territorial adult Verreaux’s eagles. Secondly, we investigate detailed movement patterns, specifically exploring the temporal (trip timing, duration, frequency and speed) and spatial (total path length and maximum distance from nest) patterns of trips away from the nest. We expect these trip parameters may change through the day due to varying lift availability for flight or energy requirements of individuals, with trips becoming longer or more frequent during times which are more energetically profitable for flying or when there is greater prey availability. Thirdly, for birds tracked in the heterogeneous Sandveld habitat, we explore habitat selection in relation to land use type and topography. In the habitat selection analysis, we expect preferential use of remaining patches of natural vegetation to exploit restricted prey resources. This research aims to provide a framework for a better understand of raptor movement ecology in order to understand space use and minimize the impacts of future human pressures and environmental change on this top predator.

## Methods

### Study Area

This study was conducted in the Cederberg and Sandveld regions in the Western Cape Province, South Africa, within the Cape Floristic Region, a recognized biodiversity hotspot [29]. The two areas have contrasting land use and topography and the eagle breeding productivity is greater in the Sandveld than the Cederberg [30]. The Cederberg mountains (elevation: 150 − 2027 m) are mostly managed by CapeNature, the statutory conservation body of the province. Within this protected area no recent land use change has occurred and there is limited human presence [31]. The vegetation type is predominantly mountain Fynbos. In contrast, the Sandveld region has little formal conservation protection and agricultural expansion has caused loss of endemic vegetation and its associated biota [32]. Following the installation of electricity distribution infrastructure in the 1980s, centre-pivot cultivation with large scale irrigation increased in this area. By the early 21^st^ century, large areas of natural vegetation was transformed to irrigated agriculture and the Sandveld was one of the most important potato (*Solanum* spp.) production areas in South Africa [33,34]. Approximately 50–70% of the area had its natural vegetation removed [33,35] and it is now characterized by a mosaic of habitats ranging from agricultural land (with no remaining natural habitat) to patches of original vegetation. Topography in the Sandveld is generally much flatter than the Cederberg, with elevations ranging from sea level up to about 1000 m.

### GPS Tracking

Adult Verreaux’s eagles were caught using Bal-chatri traps (*n* = 4) or Dho Gaza nets (*n* = 1) close to known nest sites, before breeding (*n* = 3) or during the chick rearing stage (*n* = 2), between April 2012 and April 2013 in the Cederberg (*n* = 2) and Sandveld (*n* = 3) (S1 Table). Although attempts were made to trap at similarly spaced neighbouring nests in each area, final territory selection was based on accessibility and trapping success. GPS loggers were attached using a backpack harness [36] made from 0.45” tubular Teflon Ribbon^®^ (Bally Ribbon Mills, Bally, Pennsylvania).

We used University of Amsterdam Bird Tracking System (UvA-BiTS) GPS-loggers [37], which weighed 44 g and were attached to an aluminium baseplate to aid in their fitting, bringing the total weight of the logger to 55 g (1.7% of the body weight of the lightest eagle tagged). Loggers recorded locations up to every three seconds during optimal battery conditions. However, recording frequency was dependent on solar charge and we aimed to collect data every two minutes in daylight hours. Data were downloaded in the breeding area through a ground-based antenna network or a portable base-station [37].

All tagged adult eagles were one of resident breeding pairs when trapped and all tagged eagles were from different territories. However, none of our tracked eagles completed a full year within their initial home range (see Discussion). We excluded data collected on the day of tagging from our analyses because it might not reflect normal daily activity. Days on which more than 2.5 hours of data were missing during daylight hours were also excluded. Following this, the maximum number of tracking days available per eagle for all individuals was 22 days. Therefore the data analysed in each case were limited to a subset of 22 days to allow for more direct comparisons of behaviour. Individual subsets were selected on the basis of the most comparable season available for each eagle (April − May, pre-breeding *n* = 4). Concurrent data were not available for one eagle in the Sandveld (Eagle id 723, S1 Table), which was tracked while chick rearing (Aug − Sept) and so has been excluded from all between-eagle analyses and is only included to present the comparative home range measurements and trip parameters and contribute to the habitat selection analysis. In all analyses only fixes made between sunrise and sunset (based on nautical twilight) were used. The temporal resolution of the data was standardized by sub-setting to fixes that were more than 115 seconds apart to get comparable average fix rates for all eagles (mean ± SD: 174 ± 24 s) (S2 Table).

### Home Range Analysis

We estimated home ranges as Minimum Convex Polygons (95% and 100% MCPs) using the adehabitatHR package in R v.3.0.2 [38]. Although this method is useful for exploratory purposes and comparison with other studies, it tends to overestimate the home range size and does not account for the relative intensity of space use within the total area used [39]. Due to these limitations, we also estimated home ranges, in terms of utilization distributions (UDs) using a dynamic Brownian Bridge Movement Model (dBBMM) [40]. This method quantifies the UD of an animal based on the movement path rather than individual points, by accounting for the time between locations, therefore it can process high-resolution temporally autocorrelated data [40]. dBBMM UDs were calculated using the R package ‘move’ [41]. We used a window size of 31 locations with a margin of 11 locations, which equated to a window length of one hour. The UD was mapped over a grid with cell size of 90 m. The mean positional error of UvA-BITS tags recording locations every 60 seconds is 3.23 m, this accuracy is largely due to the short interval between measurements and data collected at shorter intervals has significantly reduced the positional error [37]. Thus a location error of the GPS logger of 3 m was used in the estimations. We estimated UDs at two levels (50% and 90%) and projected home range polygons into the UTM coordinate system (WGS 1984 UTM zone 34) in QGIS (version 2.2.0) [42] for spatial mapping.

### Defining Trips from the Nest

A 400 m buffer was placed around each nest, and a ‘trip’ was defined as the eagle leaving this buffer between 07:00 and 19:00, for at least three minutes, travelling at least 1 km and excluding any overnight trips when the eagle did not return to the nest site to roost. The buffer distance was chosen based on personal observations of eagles around the nest site, which often performed display flights extending approximately this distance around the nest site. In particular, undulating display flights in the vicinity of the nest are considered to have a territorial or courtship function, as opposed to foraging trips [15]. These conditions mean that we have excluded short pseudo trips unrelated to foraging behaviour. Eagles were considered perched when the distance moved between consecutive points was less than 3 m, and these distances were zeroed in order to reduce possible error in the accumulated trip distance due to positional error in the GPS reading [43]. Total trip duration (minutes), path length (km), maximum distance from the nest (km) and average trip speed (km/h) was calculated for each trip.

To investigate when eagles were most often away on trips during the day, we calculated a measure of ‘trip probability’, which reflects the daily temporal distribution of eagle activity. This was calculated per minute of the day and is inclusive of the full duration of all active trips.

### Statistical Analysis of Trips from the Nest

All analyses were performed in R v.3.0.2. To investigate changes in trip parameters through the day of pre-breeding eagles we used Generalized Additive Models (GAM), including the start time of each trip on a decimal scale as a smooth term and ‘eagle identity’ as a fixed effect. The smooth term enables the exploration of how the trip parameters vary through the day (i.e. the start time of each trip) and enables the detection of non-linear relationships. A smooth variable can be represented by multiple splines, which are separated by knots–the edf (estimates degrees of freedom, see Results) essentially represents the flexibility of the curve, where a large edf indicates very flexible curves and an edf close to 1 indicates a near linear relationship between the test variable and the smooth term. Quasi-Poisson family was specified in all models. This assumes a Poisson error distribution, but includes a correction for data over-dispersion, which was detected in our data. For trip duration, we also tested if this was related to the residual light availability at the start time of the trip in the same fashion. Residual light availability was calculated as the time difference between trip start time and sunset (extracted per day in the Cederberg and the Sandveld using the ‘Maptools’ package in R [44]).

To compare the variability of trip parameters within an individual eagle with the variability across all eagles we tested the intraclass correlation coefficient (ICC) of each trip parameter using the ‘ICC’ package in R [45]. Temporal patterns of trip parameters were shown visually by plotting the parameters from each trip against the start time of the trip with locally weighted polynomial curves and 95% confidence intervals.

### Habitat Selection Data

We explored habitat selection for the three birds that were tracked in the Sandveld and investigated the influence of distance from the nest and topography. Habitat type was derived in QGIS from a layer that had mostly been digitized at scales 1:10,000–50:000 [46]. Four habitats were considered: natural (pristine habitat), near-natural (close to pristine), degraded (those areas which have been severely impacted but could be rehabilitated at great cost) and no natural habitat (areas which have been irreversibly transformed through development and as a result, no longer contribute to the biodiversity of the area) [31]. The topographic variables, altitude and slope, were derived from a Shuttle Radar Topography Mission (STRM) Digital Elevation Model (DEM) (approx. 30 m grid). Distance to the nest was calculated at a straight line distance (in kilometres) from the point to the nest site.

### Statistical Analysis of Habitat Selection

To investigate the locations of fixes in relation to the explanatory variables, we created a series of pseudo-absence points; these were random points generated for each eagle within its 100% MCP. For each eagle we generated 3 times as many pseudo-absence points as we had tracking fixes. We used a GLM specifying a binomial response variable (eagle points = 1; pseudo-absence points = 0) and pairwise comparisons of least-squared means. Explanatory variables entered into the GLM included distance from nest (km), habitat type (four levels), eagle identification (three levels), slope (degrees), elevation (m) and both slope and elevation squared to explore for a quadratic relationships with these terms. We tested for mulitcollinearity among variables by examining the Variance Inflation Factor (VIF). Following this, model selection was based on Akaike’s Information Criterion with a correction for finite sample sizes (AIC_c_). Models were ranked according to their AIC_c_ values and the best model was selected. The suitability and efficiency of our final model were reviewed using the Area Under the Curve (AUC) value of a Receiver Operating Characteristic (ROC) plot, which provides a measure of overall model fit [47].

## Ethics Statement

The eagle capture and tagging protocol was reviewed and approved by the Animal Ethics Committee of the Science Faculty of the University of Cape Town (reference: 2013/2012/V29/LUSM) in line with international guidelines. Following this approval, permits were issued by the provincial nature conservation body, CapeNature, authorizing the trapping and tagging of a protected species, Verreaux’s eagle, in both study areas (permits: 0035-AAA004-00744 and 0056-AAA007-00055). Private landowners in the Sandveld additionally gave permission to conduct this study. All handling, tagging and release of eagles was carried out at the site of capture to minimize duration and stress of procedures. All tags were applied with drop-off harnesses to prevent these long-lived birds from wearing them for the rest of their life.

## Results

### Home Range

**Fig 1:**
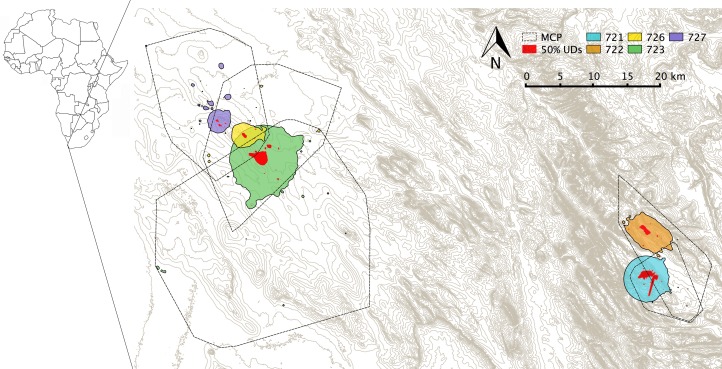
Map of home ranges for adult Verreaux's eagles in the Sandveld (*n* = 3) and the Cederberg (*n* = 2) in the Western Cape Province, South Africa. Home range estimates are given as 50% and 90% ulitisation distributions (UDs) estimated by the dynamic Brownian Bridge Movement Model (dBBMM) and Minimum Convex Polygons (100% MCPs). Red filled areas are 50% UDs; other colours are 90% UDs with the legend indicating individual eagle ID number. The Sandveld region is in the west and the Cederberg in the east of the map.

From the dBBMM UDs of the four pre-breeding eagles, we estimated average home range size (90% UD) of (mean ± SD) 27.7 ± 14.5 km^2^ and core home range (50% UD) of 1.4 ± 1.6 km^2^. All 50% UDs were small, mutually exclusive and largely centred around the nest. Average 100% MCP for pre-breeding eagles was 195.6 ± 107.2 km^2^ (Fig 1, Table 1). For another eagle (723), nesting in the Sandveld, we had information on home range size during the chick rearing period. For this bird, the three home range measures were approximately four times larger than that found for the other birds tracked during the pre-breeding period (Fig 1, Table 1).

**Table 1:** A comparison of home range estimates (km^2^) for individual resident Verreaux's eagles. 50% and 90% utilization distributions (UD) were calculated by the dynamic Brownian Bridge Movement Model [40]. Minimum Convex Polygons (MCP) and UD mean (± standard deviation) estimates are given for pre-breeding eagles only (April − May) (excluding 723 in all averages) and all eagles.

Eagle id	Status	Area	50 % UD	90 % UD	95 % MCP	100 % MCP
721	Pre-breeding	Cederberg	3.7	45.4	20.8	75.1
722	Pre-breeding	Cederberg	1.1	33.5	31.5	136.1
726	Pre-breeding	Sandveld	0.4	17.4	120.9	297.1
727	Pre-breeding	Sandveld	0.3	14.5	124.6	273.9
723	Chick rearing	Sandveld	5	112.9	102.9	775.9
		Pre-breeding	1.4 ±1.6	27.7 ±14.5	74.5 ±56.0	195.6 ±107.2
		All	2.1 ±2.1	44.7 ±40.1	80.1 ±50.1	311.6 ±275.7

### Trips from the Nest

**Fig 2:**
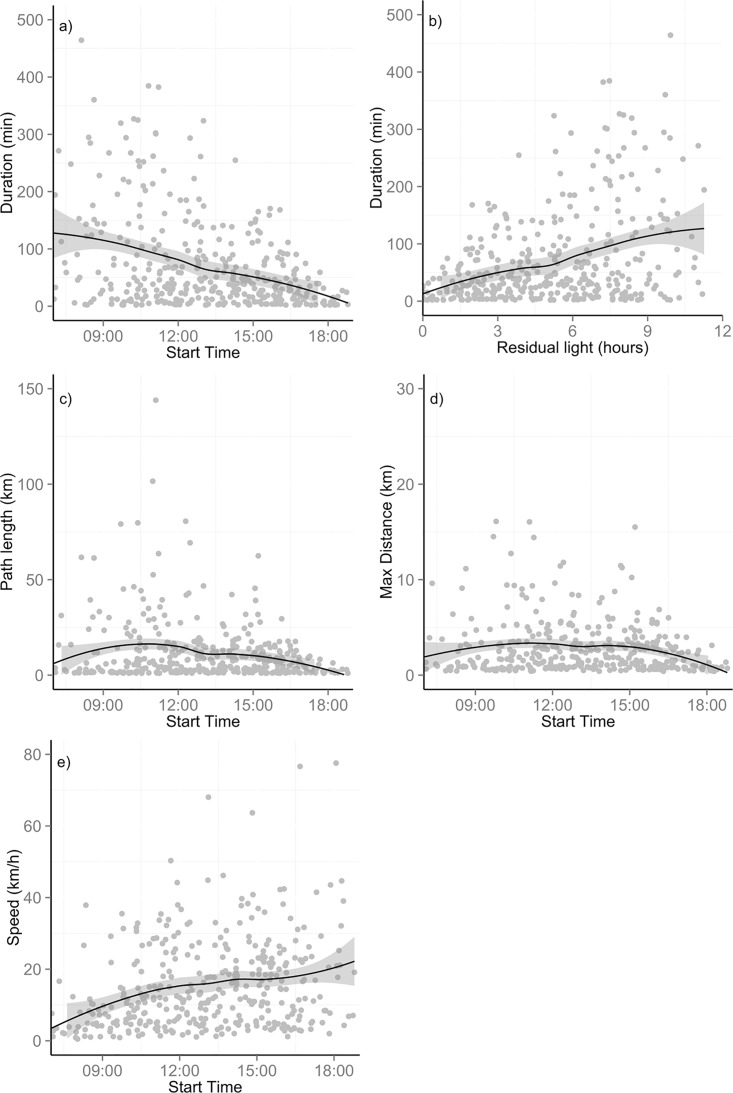
Parameters of trips from the nest by pre-breeding Verreaux's eagles (*n* = 4) over 22 days of tracking. a) Trip duration b) Trip duration regressed against residual light hours c) path length d) maximum distance travelled from the nest e) trip speed. All plotted against the time the trip was initiated (except b) and with locally weighted polynomial curves and 95% confidence interval.

During the 22 days of data we identified 369 trips from the nest by four pre-breeding eagles and an additional 174 trips by the chick rearing eagle (Table 2). Trip duration of pre-breeding eagles was found to decrease through the day (edf (estimated degrees of freedom) = 1.62, F = 31.08, *p* < 0.001; Fig 2A) and this was related to the residual light availability at the start time of the trip (edf = 1.64, F = 30.86, *p* < 0.001; Fig 2B). These birds appeared to travel longer distances in trips that were initiated just before 12:00, with path length showing a significant uni-modal relationship with time of the day (edf = 4.44, F = 5.07, *p* < 0.001; Fig 2C). Maximum distance from the nest showed similar midday peaks (edf = 3.99, F = 4.38, *p* < 0.01; Fig 2D). Trip speed increased through the day (edf = 3.25, F = 7.18, *p* < 0.001; Fig 2E), with faster trips occurring in the afternoon compared to the evening. The ICC for trip duration (ICC = −0.005) and trip distance (ICC = −0.005) was less than 1, indicating that the variability within individuals exceeded the variability across individuals. The ICC for maximum distance travelled (ICC = 0.015) and trip speed (ICC = 0.027) was low, also indicating little evidence for individual-driven variation in trip parameters.

**Table 2:** Average (mean ± standard deviation) parameters for trips from nests made by Verreaux’s eagles individually and during the pre-breeding stage (April − May) (excluding 723 in all averages) and across all eagles.

Eagle id	Path length (km)	Trip duration	Max. distance from nest (km)	Trip speed	*n*
				(km/h)	
721	11.02 ± 12.56	79 m 16 s ± 78 m 55 s	2.19 ± 1.59	11.83 ± 8.01	79
722	12.66 ± 12.80	67 m 42 s ± 78 m 07 s	3.29 ± 2.54	17.40 ± 13.44	103
726	12.02 ± 21.62	64 m 16 s ± 78 m 55 s	2.94 ± 3.41	16.34 ± 14.56	108
727	9.82 ± 13.08	72 m 21 s ± 92 m 36 s	2.74 ± 3.03	14.10 ± 10.72	79
723	12.22 ± 19.90	47 m 47 s ± 65 m 35 s	3.45 ± 4.43	20.54 ± 11.24	174
Pre-breeding	11.51 ± 15.88	70 m 10 s ± 81 m 40 s	2.83 ± 2.79	15.18 ± 12.41	369
All	11.74 ± 17.25	62 m 59 s ± 77 m 35 s	3.02 ± 3.41	16.90 ± 12.29	543

Some trip parameters did vary for the eagle that was chick rearing (eagle id: 723). This eagle tended to undertake shorter duration, faster trips, travelling further from the nest compared to the other eagles. However, the general temporal trend in trip parameters remained the same regardless of if the chick rearing eagle was included or not (S1 Fig).

**Fig 3:**
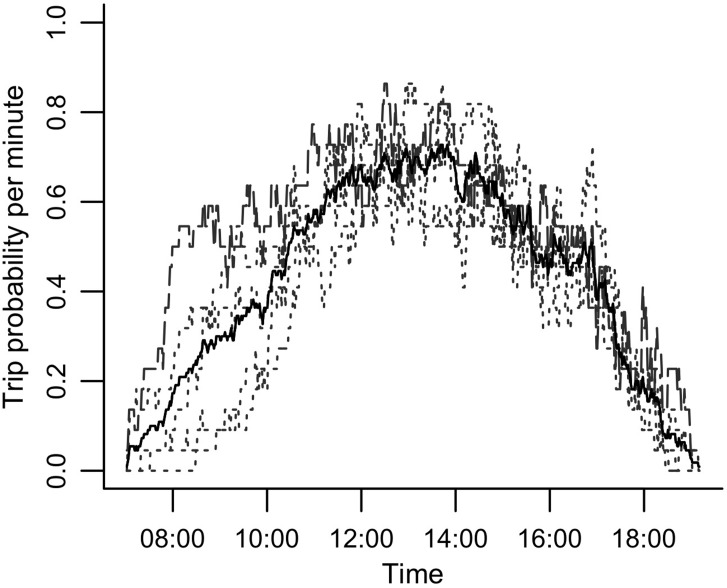
Probability of trips away from the nest for each minute of the day by Verreaux’s eagles (*n =* 5) over 22 days of tracking. The black line is the average across all eagles. Dashed line represents an eagle during the chick rearing stage, while other eagles (dotted lines) were tracked during pre-breeding stage.

The probability of an eagle being on a trip increased through the morning as more flights were initiated and declined again in the afternoon, with peak trip probability between approximately 11:00 − 15:00 (Fig 3). The chick rearing eagle had a larger trip probability than other eagles during the morning, from approximately 08:00 − 10:00 (Fig 3).

### Habitat Selection

Our most parsimonious model (*W*_*i*_ = 1) suggested that eagles responded to the topographic features elevation (elev and elev^2^) and slope (slope and slope^2^), the distance from the nest, and habitat type (S3 Table). The AUC for this model was 0.939 indicating a very good model fit.

**Fig 4:**
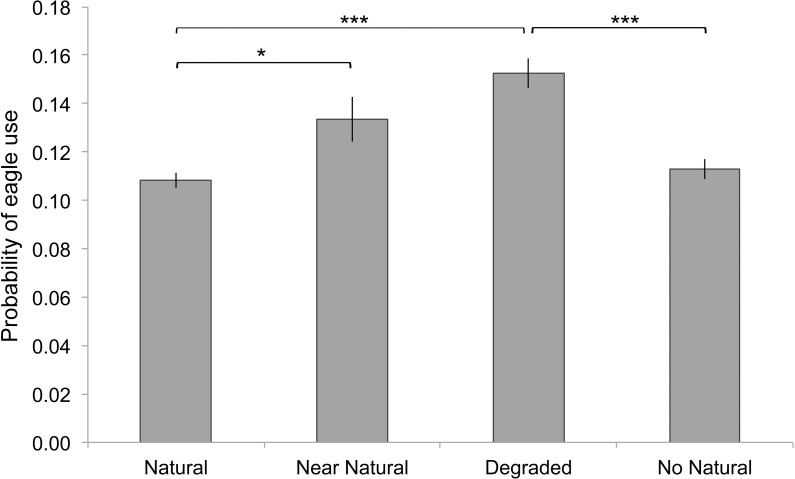
The use of four habitat types (natural, near-natural, degraded, no natural habitat) by Verreaux's eagles in the Sandveld. Habitat use takes into account distance from nest and topographic variables. * *p* < 0.05; *** *p* < 0.005 indicating significance of pairwise differences in lsmeans.

**Fig 5:**
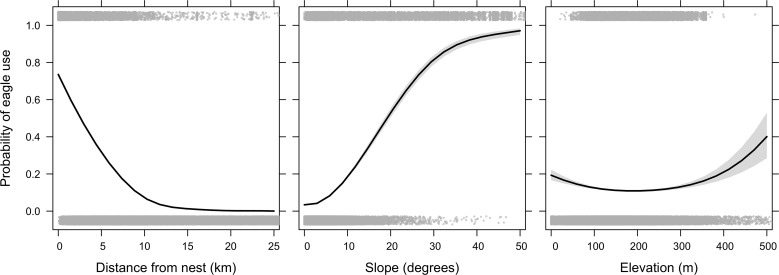
The probability of eagle use in the Sandveld in relation to distance from nest, slope and elevation (where “1” is eagle derived data and “0” is pseudo-absent points). Lines were fitted from a binomial Generalised Linear Model including and controlling for the following variables: of distance from nest (m), habitat type (four levels), slope (degrees), elevation (m) and both slope and elevation squared to explore for a quadratic relationships with these terms.

Eagles selected for intermediate habitat conditions of degraded and near-natural, over areas with natural or no remaining natural habitat as confirmed by pairwise comparisons (Fig 4, S4 Table). The probability of an area being used by an eagle within its MCP declined further away from the nest site, decreasing by 50% at 3.5 km from the nest site (Fig 5. Estimate = −0.37 ± 0.01, *z* = −76.27, *p* < 0.001). Eagle use increased with the steepness of the topographical slope (Fig 5. Estimate = 0.20 ± 0.01, *z =* 27.70, *p* < 0.01) and the quadratic term was significant although with only a small effect (Estimate = −0.0012 ± 0.0002, *z =* −5.31, *p* < 0.01). There was a quadratic relationship with elevation, whereby the probability of eagle use decreased with elevation up to about 200 m a.s.l. in a non-linear fashion (Elevation estimate = −0.0070 ± 0.0010, *z* = −6.86, *p* < 0.01, Elevation^2^ estimate = 0.000018 ± 0.000003, *z* = 6.82, *p* < 0.01). The apparent increased use of areas at higher elevations is not thought to be meaningful however, since only one eagle point occurred at elevations greater than 400 m (Fig 5).

## Discussion

Using high-resolution GPS fixes this study provides information on the spatial and temporal ranging behaviour of the Verreaux’s eagle. Although GPS data have regularly been used to investigate coarse migratory routes and home range size in raptorial species [48,49], with only a few exceptions, the use of high-resolution tracks derived from free-living raptors remains rare [50,51]. We know of no studies that have attempted to investigate accurate parameters of trips away from the nest made by any territorial raptor. This kind of information has regularly been achieved for other avian groups, notably seabirds [7,8,52], which are easier to repeatedly trap and handle and are relatively robust. Re-trapping allows download of data and recharge of batteries and thus overcomes the compromise which most raptor studies need to make between sampling frequency and study duration (as determined primarily by device weight and battery capacity) [51]. Ethical concerns are also important, owing to the fact most raptors are endangered it is not easy or ethical to frequently re-trap large numbers of raptors as researchers are able to on seabirds. Although we acknowledge that our sample size is small, owing principally to these challenges, the analyses presented in this paper were made possible by the high resolution of the data. Platform Transmitter Terminal satellite tags (PTTs) commonly used in other studies record fixes once an hour [49,53], which would not have been adequate to describe these trips given that average entire trip duration was close to one hour (i.e. 70 minutes).

Our results for the 90% UD (27.7 ± 14.5 km^2^) for pre-breeding eagles is comparable to previous estimates based on visual observations and inter-nest distances which range from 10.5–64.4 km^2^ [15,24]. The mean core home range use, considered as the 50% UD, was small (1.4 ± 1.6 km^2^), mutually exclusive and centred around the nest, typical of territorial raptors. Due to our small sample size it was not possible to compare home range sizes between seasons, and it is evident from the larger home range of the chick rearing eagle (90% UD: 112.9 km^2^) that these are likely to subject to seasonal variation. Further research should go into assessing the home range size throughout the full annual cycle. However, the mean 90% UD value represents an important area outside of the breeding season, which can contribute to conservation planning and should be considered the absolute minimum area for conservation around a nest. As a circular value this would equate to a 3 km buffer for exclusion of sensitive developments. This value is supported by the mean maximum distance from the nest (2.8 km). In the absence of any site-specific information we recommend exclusion of high intensity development, including further agricultural intensification and the placement of wind turbines, from a buffer of 3 km around any occupied nest site. However, collision risk modelling would need to take into account site specific information and the flight altitude of eagles over specific topographic features and therefore the likelihood of being in the rotor swept area [48].

Some interesting patterns were observed in the trip parameters. Trips tended to be longer in duration (but not path length) earlier in the day and the mean trip speed increased through the day. Therefore, trips in the morning were either slower or included more time spent perched compared to trips made later in the day. The decrease in trip duration throughout the day was most likely driven by the decreasing availability of daylight flying time as the day progresses, as reflected by the correlation with residual light availability. The total path length and maximum distance from nest both peaked in trips that were initiated around midday. This is likely to be related to temporal changes in lift availability, driven largely by the increased availability of thermal lift during the warmest parts of the day [54]. Raptors utilize both orographic lift and thermal lift [48,55,56] for energy-saving flight modes (such as soaring and gliding) [57,58]. The availability of orographic lift, which is generated by air movements over steep slopes and cliffs, is independent from time of day and largely dependent on wind and topographic conditions [55,59]. However, thermal lift is generated by solar radiation heating the ground and then heating patches of air causing rising pockets of warmer lower density air. Therefore, thermal lift develops in strength and intensity with increasing temperature in the day. The centring of peak trip probability around midday is also likely to be caused by a reliance on thermals for flight activity. This use of thermals could allow for longer distances to be travelled while not increasing trip duration as the use of thermals allows faster flight speed than the use of orographic lift [55]. However, we cannot exclude the possibility that these patterns could be associated with temporal patterns in prey behaviours, which we do not have information on [16].

We attribute the larger trip probability for the chick rearing eagle compared to pre-breeding eagles during the morning (08:00 − 10:00) to the need to provision food to the chick. This increase in activity between seasons is unlikely to be caused by greater thermal lift availability because chick rearing occurs during the austral winter when mornings are colder than the pre-breeding period. Although the chick rearing eagle made trips of similar path length compared to other eagles, these trips were faster, resulting in more frequent, shorter duration trips which started earlier in the day, probably also owing to the necessity to provision food. Similar increases in the amount of time spent flying during this period, particularly by males, have been noted in other raptors [60].

Habitat selection within agriculturally transformed landscapes has been examined in various raptorial species including golden eagle *Aquila chrysaetos* [61], Spanish imperial eagle *Aquila adalberti* [62], marsh harrier *Circus aeruginosus* [63] and hen harrier *Circus cyaneus* [6], where preferences are usually determined by food abundance or availability. Despite our small sample size (*n =* 3 eagles) and relatively short duration of the study (22 days), within the Sandveld we were able to use the high-resolution tracking data (*n =* 12,506 eagle locations) to build habitat selection models describing space and habitat use by three eagles. Unsurprisingly, given the territorial nature of the species and the fact that they are central place foragers [64], we found that eagles were more likely to use areas closer to the nest than further away. Eagles were also found to select steeper areas, which likely reflects their tendency to perch on cliffs or the use or slopes for orographic lift [56]. A selection preference was also shown for lower elevation topography, which is potentially related to food availability or a tendency for better thermal lift availability over such areas [65]. Controlling for these important constraining variables (i.e. nest distance and topography), the habitat selection analysis showed a preference for intermediately converted habitat types (i.e. near-natural and degraded), suggesting that Verreaux’s eagles may be more resilient to a certain level of agricultural transformation than previously thought. Foraging theory predicts that raptors will forage preferentially in patches offering highest net energy gain [66]. This may be determined by prey abundance or availability, whereby prey availability to a predator can be determined by the physical accessibility in different habitat structures [67]. Although we have no information on prey abundance in these different habitats, near-natural and degraded habitats are likely to feature intermediate levels of ground cover, and offer more visible or accessible hunting opportunities while still maintaining an adequate prey base. The use of transformed habitats in the Sandveld is reflected by the varied diet composition of Verreaux’s eagles in this area. In particular, it is likely that a large proportion of the mole-rats (*Bathyergus suillus*, *B*. *janetta*) consumed by eagles are obtained in these transformed habitats [68]. This habitat selection could also contribute to explaining the higher than average breeding productivity of Verreaux’s eagles in the Sandveld region [30]. The less intensive use of areas with no natural habitat, suggests that the suitability of the habitat mosaic for Verreaux’s eagles is delicately balanced, and further agricultural development could reduce the suitability of the area [68]. This reflects patterns seen in other systems, whereby some agricultural transformation may benefit avifauna [63,69–71] but beyond a certain threshold level of habitat loss the balance is tipped and can lead to rapid species declines or loss [72]. For example, in lesser kestrels *Falco naumanni* low level agricultural transformation has been associated with an increase in breeding productivity [73]. However, beyond a threshold of habitat conversion for agriculture, suitable foraging habitat and an adequate prey base are not maintained potentially resulting in reduced reproductive output, such as that seen in Eurasian kestrels *Falco tinnunculus* [74].

The effects of transmitters on the breeding productivity, longevity and behaviour of raptors should be subject to further consideration and research [75,76]. The duration of the present study was curtailed by the loss of three eagles within the first 40 days of tagging. One male eagle was found dead 21 km away from its nest (S1 Table, Eagle id 723). However, we were unable to locate it for nearly a year post-mortem therefore the cause of death remains unknown, there is a possibility it was ousted from its territory prior to death, because a replacement bird was observed on its territory immediately after its disappearance. Another two deaths were believed to be due to intra-specific conflict; two neighbouring tagged female eagles (S1 Table, Eagle id 726 and 727) are believed to have killed each other, suggested by our GPS tracking data revealing they were at the same location for a few minutes before returning to the vicinity of their respective nests and subsequently going offline permanently. In addition, one eagle (S1 Table, Eagle id 721) was ousted from its territory and although it survived, further contact was intermittent because of its massively expanded range and the constraints of the download system [37,77]. We are uncertain if this seemingly high turnover of adults was the product of a natural process, perhaps driven by a large population of ‘floaters’ (unpaired eagles) in the two areas, or was caused by the physical effect of the transmitters on the study birds. Although Verreaux’s eagles are generally considered to be monogamous, no adequate colour marking or DNA study has ever been undertaken to confirm this and potentially mate changes may be more frequent that believed. Increased energetic costs [78], reduced breeding attempts [79,80] and reduced body condition [81] have all been related to the use of GPS transmitters or telemetry devices on birds. However, recent studies on *Aquila* eagles using these techniques have not reported abnormal responses to tagging in adult or juvenile birds [3,4,82]. Behavioural or ecological responses to tagging are often difficult to detect and we recommend continued reporting of potential costs to study birds and cost-benefit considerations in future tagging studies.

Sex or study area were not used as explanatory variables for any of these analyses although they should be accounted for, particularly as in this case these variables are mostly confounded with each other. However, a study of golden eagle ranging behaviour found that males and females travelled similar distances [61] and observations of Verreaux’s eagles indicate that they spend 95% of the day together outside of the breeding season [15]. Currently, the major limitation of this study remains the small sample size of eagles. Although caution is required when interpreting these results because; (i) they are based on a limited sample size of birds and (ii) dissimilarities are present between the contrasting study areas, primarily topography, which is likely to contribute to overall energy expenditure [57] and could obscure movement parameters, we believe that it is unlikely that a larger data set would vastly change our conclusions although a larger sample size would allow the identification of seasonal or sex-based movement trends. Despite these limitations this research provides the first framework for a better understanding of fine-scale raptor movement ecology and enhances our understanding of ranging behaviour of a top predator. In today’s rapidly changing environment, biodiversity is increasingly exposed to anthropogenic threats. Studies such as this will be particularly relevant to resolving conflict between raptors and land-use change, habitat loss and other developments within close proximity of nest sites [83].

## Supporting Information

S1 Data Trips from nests data. (XLSX)

S2 Data Habitat selection analysis data. (XLSX)

S1 Fig Parameters of trips from the nest by Verreaux's eagles (*n* = 5, including one chick-reading eagle) over 22 days of tracking. a) Trip duration b) path length c) maximum distance travelled from the nest d) trip speed. All plotted against the time the trip was initiated and with locally weighted polynomial curves and 95% confidence interval.
(TIF)

S1 Table Summary of all Verreaux's eagles tracked and the GPS fixes obtained. (DOCX)

S2 Table Summary of the tracking data subsetted for use in the analysis of Verreaux's eagle ranging behaviour. (DOCX)

S3 Table Results from the top five GLMs comparing model fit for habitat selection of Verreaux’s eagles. Model parameters abbreviations: elev, elevation; elev^2^ and slope^2^ for quadratic inference; egl_id, individual eagle id; hab_typ, habitat type; nest_dist, distance from nest. Other column abbreviations: df, degrees of freedom; logLik, log likelihood; ΔAICc, change in AICc relative to the highest ranked model; *wi*, AICc weight. The top model is shown in bold.
(DOCX)

S4 Table Model coefficients of the GLM analysing habitat selection of Verreaux’s eagles in the Sandveld region of South Africa. (DOCX)
