# Supplementary figures and images for: Ranging Behaviour of Verreaux’s Eagles during the Pre-Breeding Period Determined through the Use of High Temporal Resolution Tracking
Source: PLoS One. 2016 Oct 10;11(10):e0163378. doi: 10.1371/journal.pone.0163378 (PMC5056708; doi:10.1371/journal.pone.0163378)

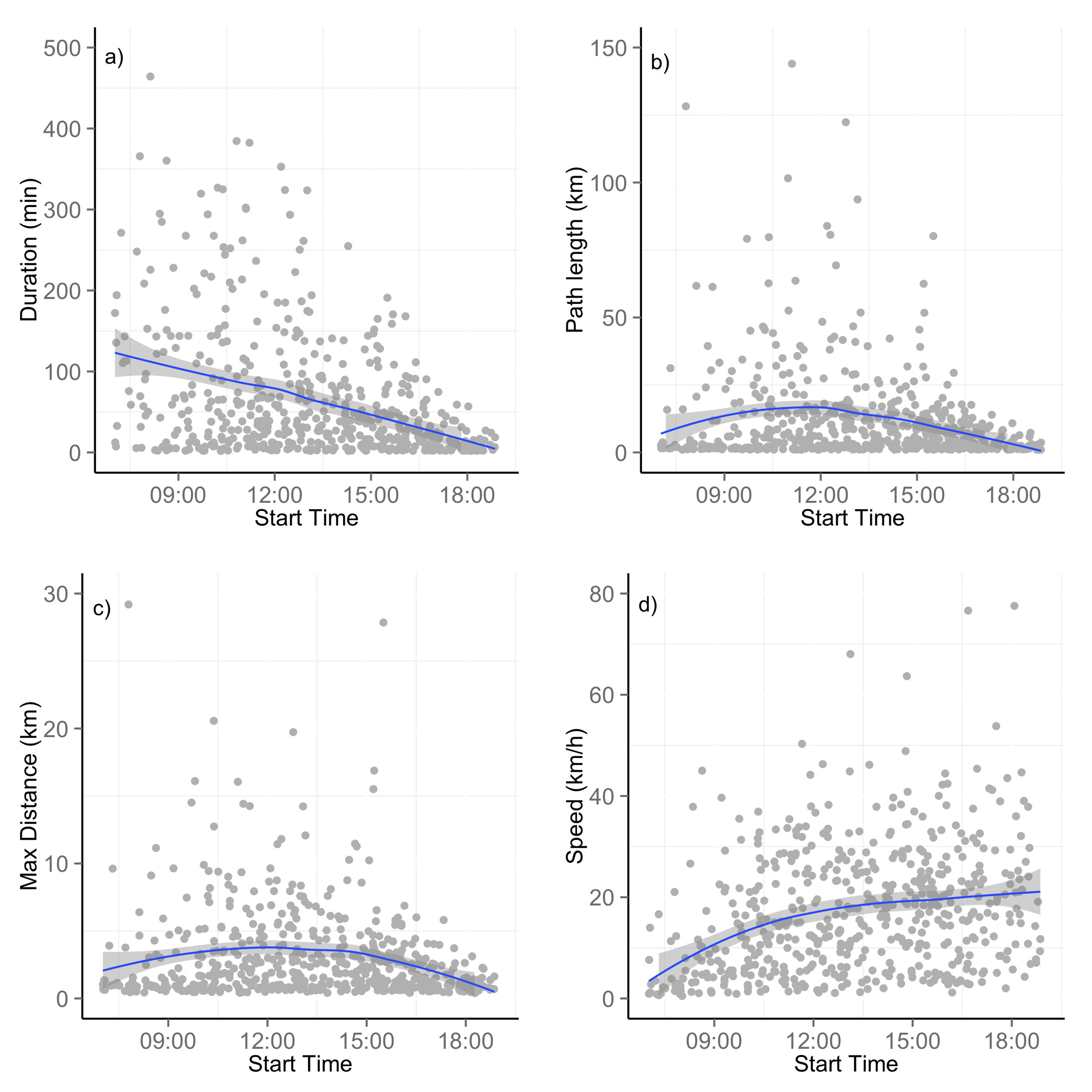

Supplement: S1 Fig — a) Trip duration b) path length c) maximum distance travelled from the nest d) trip speed. All plotted against the time the trip was initiated and with locally weighted polynomial curves and 95% confidence interval. (TIF) [file pone.0163378.s003.tif]
