# Supplementary material for: Ranging Behaviour of Verreaux’s Eagles during the Pre-Breeding Period Determined through the Use of High Temporal Resolution Tracking
Source: PLoS One. 2016 Oct 10;11(10):e0163378. doi: 10.1371/journal.pone.0163378 (PMC5056708; doi:10.1371/journal.pone.0163378)
Supplement: S1 Table — (DOCX) [file pone.0163378.s004.docx]

S1 Table. Summary of all Verreaux's eagles tracked and the GPS fixes obtained.

| Eagle id | Study area | Sex | Tag date | End track | Eagle fate | *n* total fixes |
| --- | --- | --- | --- | --- | --- | --- |
| 721 | Cederberg | male | 06/04/2012 | 10/07/2013 | ousted | 117839 |
| 722 | Cederberg | male | 26/09/2012 | 11/05/2013 | unknown | 393926 |
| 723 | Sandveld | male | 25/08/2012 | 11/09/2012 | died | 31144 |
| 726 | Sandveld | female | 10/04/2013 | 18/05/2013 | killled in territorial dispute | 14643 |
| 727 | Sandveld | female | 13/04/2013 | 18/05/2013 | killled in territorial dispute | 10538 |
