# Supplementary material for: Ranging Behaviour of Verreaux’s Eagles during the Pre-Breeding Period Determined through the Use of High Temporal Resolution Tracking
Source: PLoS One. 2016 Oct 10;11(10):e0163378. doi: 10.1371/journal.pone.0163378 (PMC5056708; doi:10.1371/journal.pone.0163378)
Supplement: S2 Table — (DOCX) [file pone.0163378.s005.docx]

S2 Table. Summary of the tracking data subsetted for use in the analysis of Verreaux's eagle ranging behaviour.

| Eagle id | Status | Date range | *n* fixes | *n* daylight fixes | mean daylight fix interval | mean fixes per day |
| --- | --- | --- | --- | --- | --- | --- |
| 721 | Pre-breeding | 10/04/2012 − 08/05/2012 | 6231 | 4888 | 2 min 47 sec | 222 |
| 722 | Pre-breeding | 11/04/2013 − 03/05/2013 | 6407 | 5693 | 2 min 25 sec | 259 |
| 726 | Pre-breeding | 11/04/2013 − 03/05/2013 | 5186 | 4245 | 3 min 24 sec | 193 |
| 727 | Pre-breeding | 14/04/2013 − 07/05/2013 | 5579 | 4644 | 3 min 12 sec | 211 |
| 723 | Chick rearing | 26/08/2012 − 20/09/2012 | 6341 | 5529 | 2 min 41 sec | 251 |
