# Supplementary material for: Ranging Behaviour of Verreaux’s Eagles during the Pre-Breeding Period Determined through the Use of High Temporal Resolution Tracking
Source: PLoS One. 2016 Oct 10;11(10):e0163378. doi: 10.1371/journal.pone.0163378 (PMC5056708; doi:10.1371/journal.pone.0163378)
Supplement: S3 Table — Model parameters abbreviations: elev, elevation; elev2 and slope2 for quadratic inference; egl_id, individual eagle id; hab_typ, habitat type; nest_dist, distance from nest. Other column abbreviations: df, degrees of freedom; logLik, log likelihood; ΔAICc, change in AICc relative to the highest ranked model; wi, AICc weight. The top model is shown in bold. (DOCX) [file pone.0163378.s006.docx]

**S3 Table. Results from the top five GLMs comparing model fit for habitat selection of Verreaux’s eagles.** Model parameters abbreviations: elev, elevation; elev^2^ and slope^2^ for quadratic inference; egl_id, individual eagle id; hab_typ, habitat type; nest_dist, distance from nest. Other column abbreviations: df, degrees of freedom; logLik, log likelihood; ΔAICc, change in AICc relative to the highest ranked model; *wi*, AICc weight. The top model is shown in bold.

| Model | df | logLik | ΔAIC_c_ | *W_i_* |
| --- | --- | --- | --- | --- |
| **Elev+elev2+slope+slope2+hab_type+nest_dist+egle_id** | **11** | **-14159.18** | **0** | **1** |
| Elev+elev2+slope+hab_type+nest_dist+egle_id | 10 | -14171.59 | 22.81 | 0 |
| Slope+slope2+hab_type+nest_dist+egle_id | 9 | -14181.74 | 41.1 | 0 |
| Elev+slope+slope2+hab_type+nest_dist+egle_id | 10 | -14181.19 | 42.01 | 0 |
| Slope+hab_type+nest_dist+egle_id | 8 | -14193 | 61.63 | 0 |
| Elev+elev2+slope+slope2+nest_dist+egle_id | 8 | -14193.54 | 62.71 | 0 |
