# Supplementary material for: Ranging Behaviour of Verreaux’s Eagles during the Pre-Breeding Period Determined through the Use of High Temporal Resolution Tracking
Source: PLoS One. 2016 Oct 10;11(10):e0163378. doi: 10.1371/journal.pone.0163378 (PMC5056708; doi:10.1371/journal.pone.0163378)
Supplement: S4 Table — (DOCX) [file pone.0163378.s007.docx]

S4 Table. Model coefficients of the GLM analysing habitat selection of Verreaux’s eagles in the Sandveld region of South Africa.

|  |  |  |  |  | Confidence intervals | |
| --- | --- | --- | --- | --- | --- | --- |
|  | Estimate | Std. Error | z value | P | 2.5 % | 97.5 % |
| (Intercept) | 1.37 | 0.12 | 11.83 | < 2 x 10^16^ | 1.14 | 1.59 |
| Natural | -0.39 | 0.05 | -8.07 | 7.14 x 10^16^ | -0.49 | -0.30 |
| Near Natural | -0.16 | 0.09 | -1.80 | 0.07 | -0.33 | 0.01 |
| No Natural | -0.35 | 0.05 | -6.50 | 8.27 x 10^11^ | -0.45 | -0.24 |
| Nest distance | -0.36 | 4.70 x 10^3^ | -76.27 | < 2 x 10^16^ | -0.37 | -0.35 |
| Slope | 0.20 | 0.01 | 27.70 | < 2 x 10^16^ | 0.18 | 0.21 |
| Slope^2^ | -1.21 x 10^3^ | 2.28 x 10^4^ | -5.31 | 1.09 x 10^7^ | -1.64 x 10^3^ | -7.51 x 10^4^ |
| Elevation | -0.01 | 1.02 x 10^3^ | -6.86 | 6.94 x 10^12^ | -0.01 | -4.96 x 10^3^ |
| Elevation^2^ | 1.81 x 10^5^ | 2.65 x 10^6^ | 6.82 | 8.84 x 10^12^ | 1.28 x 10^5^ | 2.32 x 10^5^ |
| eagle_id726 | -1.35 | 0.04 | -34.09 | < 2 x 10^16^ | -1.43 | -1.28 |
| eagle_id727 | -1.34 | 0.04 | -34.98 | < 2 x 10^16^ | -1.41 | -1.26 |
